# Neoadjuvant Chemotherapy for Different Stages of Muscle-Invasive Bladder Cancer: A Systematic Review and Meta-analysis

**DOI:** 10.1155/2022/8493519

**Published:** 2022-03-02

**Authors:** Shuai Liu, Yu Yao, Fengju Guan, Lijiang Sun, Guiming Zhang

**Affiliations:** Department of Urology, The Affiliated Hospital of Qingdao University, Qingdao, China

## Abstract

The purpose of this meta-analysis is to determine the survival benefits and pathological outcomes of neoadjuvant chemotherapy (NAC) combined with radical cystectomy (RC) administered to patients with cT2 or cT3-4N0M0 muscle-invasive bladder cancer (MIBC). PubMed, Embase, and the Cochrane Library were searched for comparing the use of NAC in combination with RC and RC alone in patients with different MIBC stages. A fixed effects model was used to calculate hazard ratio (HR) and odds ratio (OR) with 95% confidence intervals (CIs), and the *I*^2^ statistic was used to assess heterogeneity. Moreover, we determined possible sources of heterogeneity by subgroup and sensitivity analyses. Fifteen studies were finally selected. For cT2 bladder cancer, NAC combined with RC significantly increased the rates of pathological complete response (pCR) (OR = 4.84, 95% CI: 1.18–19.92, *p* = 0.029) but did not improve overall survival (OS) (HR = 0.86, 95% CI: 0.72–1.02, *p* = 0.078) across six studies. Regarding cT3-4 bladder cancer, NAC has a significantly improved effect on OS (HR = 0.69; 95% CI: 0.59–0.81, *p* < 0.001, across seven studies and 5726 patients) and pCR (pooled OR = 4.80; 95% CI: 2.06–11.23, *p* < 0.001, across two studies) than RC alone. Most studies were randomized prospective trials (level 1 evidence), and all the effects were irrespective of the type of study design and did not vary between subgroups of patients. In conclusion, NAC combined with RC is recommended for patients with T3-4aN0M0 but not for patients with T2N0M0.

## 1. Introduction

As the 10th most common malignancy, the incidence of bladder cancer (BC) in males is much higher than that in females [1]. Southern and Western Europe as well as North America has the highest incidence of BC in the world [1]. About one quarter of the newly diagnosed BC were found to invade the muscle tissue. Although radical cystectomy (RC) is widely used currently, distant metastases still occur in half of the patients after surgery [2]. In the 1970s, the effectiveness of chemotherapeutic drugs to treat BC was determined, representing a milestone that led to the beginning of neoadjuvant cisplatin-based chemotherapy, because of the obviously improved survival for the first time in decades [3, 4]. Consequently, a combination of systemic chemotherapy with locally surgical removal plays a critical role in decreasing disease recurrence.

cT2 BC is an organ-confined disease that indicates a lower risk of localized progression and distant metastases. Although with constant attempts, there is not yet a consensus on the optimum strategy for patients with cT2 BC. Neoadjuvant cisplatin-based chemotherapy combined with RC may improve the prognosis for patients with BC [5–7].

Administration of neoadjuvant chemotherapy (NAC) before RC has been reported to improve survival rates lead to pathological downstaging and enhance pathological complete response (pCR) compared with RC alone [8–10]. Moreover, Sherif et al. found a significantly improved survival rate in cT3 stage only [11]. However, no statistically significant difference in overall survival in the cT2 group was observed between NAC plus RC and RC alone. The results of NAC may change with differences between stage groups when all stages are included [8, 12, 13]. The value of NAC plus RC administered to patients with different staging of muscle-invasive BC (MIBC) has been the subject of numerous trials, although there is not enough information to systematically assess its pathological and clinical benefits.

The survival benefit of NAC combined with RC for patients with cT2–4N0M0 disease has been established [9]. However, there are many side effects for NAC, such as neurotoxicity, nephrotoxicity, and hearing loss, and it can lead to cardiac dysfunction [6, 14]. The use of NAC in cT2 disease may be overtreatment and result in unnecessary side effects. Previous studies reported that NAC followed by RC did not bring survival benefits for patients with cT2 disease [11, 15–24], but no meta-analysis was performed. The purpose of this meta-analysis was to assess the efficacy of preoperative NAC on the survival outcomes of patients with cT2 or cT3-4 disease.

## 2. Materials and Methods

### 2.1. Search Strategy and Selection Criteria

This systematic review and meta-analysis was performed following PRISMA guidelines [25]. We searched the Cochrane Central Register of Controlled Trials (CENTRAL), PubMed, and Embase to pinpoint observation cohort studies and randomized controlled trials (RCTs) published from inception to September 2021. Only papers written in English were included. We used the following search strategy: (“bladder”) AND (“neoadjuvant” OR “neo-adjuvant” OR “neo adjuvant”). The details are provided in Supplementary material 1. When studies were conducted in the same centers and the time overlapped, we adopted the latest study. Two reviewers independently reviewed the search results. Once there were disagreements, a third reviewer evaluated and solved them.

We assessed RCTs or cohorts using the predetermined PICOS way as the inclusion and exclusion criteria. PICOS approach refers to P—population, I—intervention, C—comparison, O—outcome, and S—study design. We applied the following selection criteria: (P) patients with a different stage of MIBC; (I) patients who underwent NAC plus primary therapy (RC); (C) patients who underwent RC alone; (O) cancer-specific survival (CSS), overall survival (OS), pathological downstaging (pT_DS; pT staging < cT staging), pathological partial response (pPR; pT staging < ypT2N0M0), and pathological complete response (pCR; pT stage ≤ ypT0N0M0); and (S) retrospective or prospective studies. We excluded studies that assessed upper urinary tract urothelial carcinoma and did not clearly state or define results.

### 2.2. Data Extraction and Quality Assessment

The following data were extracted from the selected papers: year, first author, country, type of study, participant population, cancer stage, chemotherapeutic drugs, follow-up duration, and results (hazard ratios (HRs), 95% confidence intervals (CIs), and number of events reported in the article). The NAC regimen comprised gemcitabine and cisplatin (GC), methotrexate, vinblastine, doxorubicin, and cisplatin (MVAC), or other cisplatin-based therapies. We found that participants in a Swedish research [22] were incorporated in another Northern Europe multicenter research [11] and participants in two American research [20, 21] were incorporates in another American multicenter research as well [16]. In consequence, we included studies with higher quality and more participants into meta-analysis in order to avoid repetitive participants. The risk of bias of the RCTs was assessed via the Cochrane Collaboration risk of bias [26]. We evaluated the quality assessment of nonrandomized controlled trials via the Newcastle–Ottawa Quality Assessment Scale [27]. Two reviewers independently performed data extraction and evaluation of the study quality. Differences were raised and solved by a third reviewer.

### 2.3. Data Synthesis and Analysis

For survival results, we extracted or calculated HRs and 95% CIs for randomized and nonrandomized controlled trials. When the HR and 95% CI of the research could not be obtained, we adopted a widely quoted method that provided the way for computing time-to-event data based on existing information [28]. For pathological outcome, we extracted the total number of patients and the number of pathological complete response to calculate the ORs and corresponding 95% CIs. We evaluated the degree of heterogeneity by calculating the *I*-squared statistic and the Cochran Q statistic [29]. *Q* test (*p* > 0.1) and *I*^2^ < 50% represent no significant heterogeneity. When the heterogeneity is not significant, we chose to use the fixed effects model. Otherwise, the random effects models were used [30]. If heterogeneity was substantial, by conducting subgroup analyses or sensitivity analyses, the possible cause of heterogeneity would be investigated. We used Stata software (version 16.0) for all statistical analyses.

## 3. Results

### 3.1. Study Selection and Characteristics

**Figure 1:**
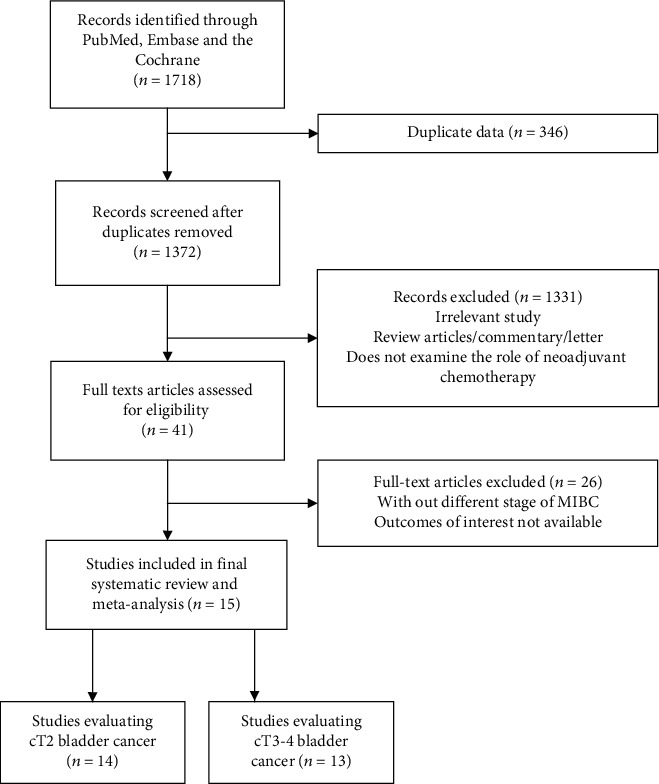
Preferred Reporting Items for Systematic Reviews and Meta-analysis (PRISMA) flowchart.

In all, 1718 candidate papers were identified using the search formula. Then, 41 studies were extracted based on the title or abstract. Thereafter, we performed a qualitative analysis of each article and reduced the included articles to the last 15, among which 14 analyzed cT2 BC and 13 analyzed cT3-4 BC (Figure 1). Nine studies divided the stages into cT2 and cT3-T4, and three studies separately listed cT2, cT3, and cT4 disease. Two studies analyzed cT2 only, and one study analyzed cT3 only. Seven prospective and eight retrospective studies were included in our study, including 13971 patients. The follow-up period ranged from 1.5 years to 9.8 years. RC was performed in 11 trials [16–24]. Three trials performed preoperative radiotherapy and cystectomy [11]. Radical radiotherapy alone is out of date in the treatment of BC, and therefore, this study was excluded [15]. Most trials used cisplatin combined with several other chemotherapeutic drugs, such as gemcitabine, vinblastine sulfate, and doxorubicin hydrochloride. One study included a variety of regimens, such as carboplatin regimen and cisplatin regimen [23]. NAC regimen was not mentioned in 4 studies [19, 21, 22, 24]. Cisplatin doses were 70 mg/m^2^ every cycle for 2 cycles (two trials) or for 3 cycles (four trials). The detailed characteristics of 15 included studies are given in Table 1, and Supplementary Figure 1 and Supplementary Table 1 demonstrate the results of risk of bias assessment.

### 3.2. Results of cT2 Staging

Fourteen studies evaluated the effect of NAC in cT2 BC. They had a comparison arm with RC alone, of which six were prospective and eight were retrospective. To avoid duplication of patients, six studies were finally included. All of these studies used platinum-based combinations. Five studies used RC as the local treatment, and one study used preoperative radiation plus cystectomy or cystectomy.

**Figure 2:**
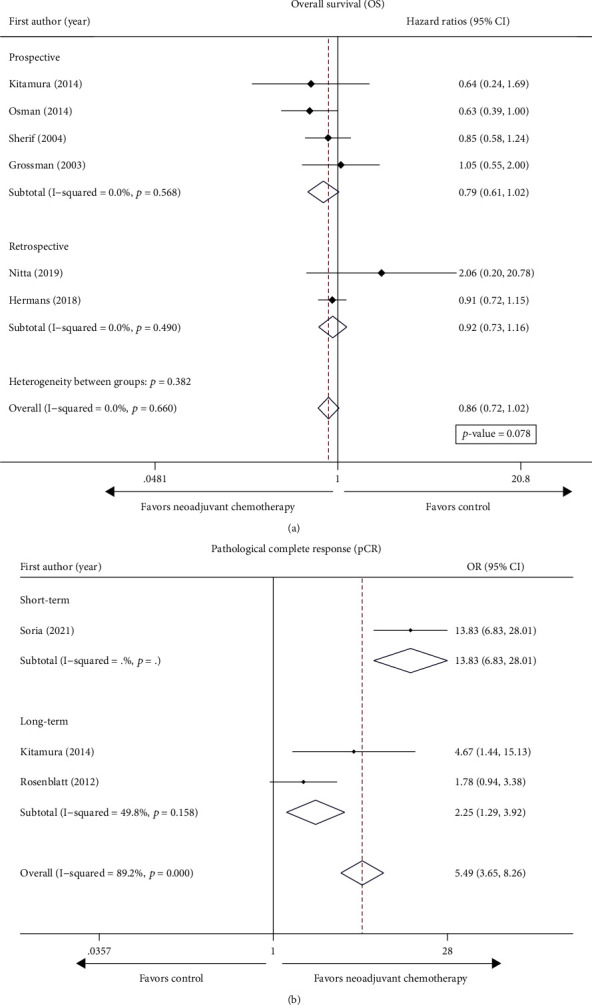
Forest plot of studies evaluating the efficacy of neoadjuvant chemotherapy for cT2 on (a) overall survival (OS) and (b) pathological complete response (pCR).

The survival results were indicated below. Six comparative trials comprised a total of 5612 patients. OS was the reported survival outcome. Surprisingly, we showed that the use of NAC followed RC did not bring survival benefit. OS of patients with cT2N0M0 BC (HR = 0.863, 95% CI: 0.727–1.025, *p* = 0.078) was not statistically different with no heterogeneity (*I*^2^ = 0.0%, *p* = 0.66), and results are given in Figure 2(a). We used a fixed effects model because there was no heterogeneity.

The pooled results of pathological outcomes were as follows. Overall, pCR was achieved in 137 of 440 (31.1%) patients after neoadjuvant treatment and in 36 of 445 (19.0%) in the platinum-free chemotherapy group. Here, we demonstrated that NAC before RC significantly increased the rates of pCR (ypT0N0) (OR = 4.84, 95% CI: 1.18–19.92, *p* = 0.029). The heterogeneity is substantial (*Q* test *p* < 0.001 and *I*^2^ = 89.2%); therefore, we adopted a random effects model.

Considering the difference of study designs, subgroup analysis was performed to assess the reliability of the outcomes. Consistent with the pooled results, NAC before RC showed no significant effect of overall survival in the prospective (HR, 0.79; 95% CI: 0.61–1.02, *I*^2^ = 0.0%) and cohort studies (HR, 0.92; 95% CI: 0.73–1.16, *I*^2^ = 0.0%), as shown in Figure 2(a). We only observed heterogeneity in the pathological results of cT2 staging (Cochran *Q* statistic *p* < 0.001 and *I*^2^ = 89.2%). Consistent with the pooled results, NAC before RC significantly increased the rates of pCR (ypT0N0) in the long-term follow-up (OR = 2.24, 95% CI: 1.29-3.92, *I*^2^ = 49.8%) and the short-term follow-up (OR = 13.83, 95% CI: 6.83-28.01) (Figure 2(b)).

### 3.3. Results of cT3-4 Staging

Seven prospective and 6 retrospective trials explored the impact of NAC plus RC in patients with cT3-4 (Table 1). Two studies presented the survival results of cT3 and cT4 separately, and the other studies presented the results of cT3-4.

**Figure 3:**
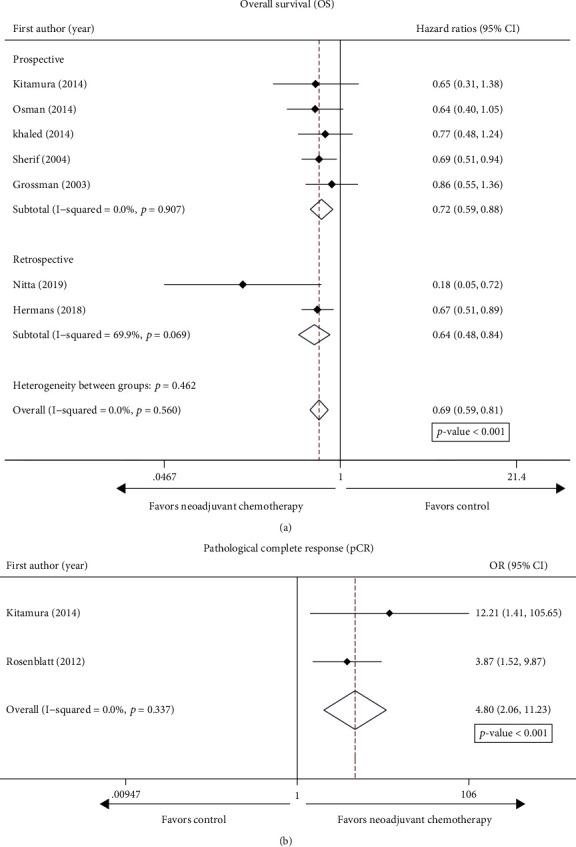
Forest plot of studies evaluating the efficacy of neoadjuvant chemotherapy for cT3-4 on (a) overall survival (OS) and (b) pathological complete response (pCR).

The survival results were indicated as follows. A total of 7 studies were eligible for the final analysis. We found that NAC before local treatment significantly prolonged the OS of patients with cT3-4N0M0 BC (pooled HR = 0.69, 95% CI: 0.59–0.81) with low heterogeneity (*I*^2^ = 0.0%, *p* = 0.56), as shown in Figure 3(a). With a median follow-up of 4.7 years, the RCT by Sherif et al. validated that NAC played a remarkable role in the OS of patients with cT3-4N0M0 BC (HR = 0.69, 95% CI: 0.51–0.94). Similarly, the other two cohort trials by Nitta et al. and Hermans et al. found that NAC before RC improved the outcome of OS (HR = 0.67, 95% CI: 0.51–0.89; HR = 0.18, 95% CI: 0.05–0.72). The randomized JCOG0209 trial performed by Kitamura et al. showed that NAC did not bring obvious benefit of OS for cT3-4N0M0 BC (HR = 0.65, 95% CI: 0.31–1.38). RCTs by Osman et al. and Grossman et al. found no statistically significant difference of NAC on OS for cT3-4N0M0 BC (HR = 0.645, 95% CI: 0.395–1.054; HR = 0.86, 95% CI: 0.55–1.36).

For cT3-4 patients, overall, the pT0 proportions in NAC groups and the control groups were 19.4% and 4.8%, respectively. NAC plus RC had a remarkable effect on the achievement rates of the pCR (ypT0N0) in comparison with the comparison groups for the cT3–4 BC (OR = 4.80, 95% CI: 2.06–11.23, *I*^2^ = 0.0%), as is illustrated in Figure 3(b).

By the same token, the subgroup analysis of study design was performed. It showed that the survival and pathological outcomes were consistent with the above results (Figure 3(a)). NAC plus RC significantly improved overall survival compared with RC alone.

## 4. Discussion

RC has been considered the standard management for MIBC patients all the time. In recent years, NAC has been recommended for MIBC disease by most urologists and oncologists. A large number of studies have confirmed that NAC for MIBC could control tumor progression, shrink tumor size, and reduce the rate of distant metastasis [11, 15–24, 36]. However, whether NAC may bring the same benefits for patients with T2, T3, or T4N0M0 MIBC separately is still doubtful. To address this question, we conducted this meta-analysis. Although NAC before RC significantly prolonged OS for patients with MIBC, no significant improvements in OS for patients with T2N0M0 were recognized when NAC before RC was compared with RC alone.

The meta-analysis conducted by the Advanced Bladder Cancer (ABC) Meta-analysis Collaboration of randomized trials of patients with invasive BC revealed that platinum-based NAC before RC confers a remarkable improvement in oncological outcomes, which is associated with better OS and disease-free survival. Therefore, NAC plus RC has been widely used as a treatment code for BC with myometrial invasion [37, 38]. Several meta-analyses [12, 13] reported the same results that participants administrated with NAC plus RC had a significant difference in longer survival time than those with RC only, whereas a literature review [39] showed that OS did not differ between NAC plus RC groups and RC only groups. However, single-agent platinum was included in the study [39] without subgroup analyses and may explain this discrepancy. Several randomized-controlled trials (BA06 30894, SWOG-8710, and Nordic I-II) have demonstrated the effect of NAC to inhibit tumors, with an approximately 6%-8% improvement in five-year survival rate.

All staging of MIBC was included in most current studies at the same time. It may be inaccurate to analyze the results of all staging of MIBC together. Consequently, we only selected trials that analyze the results of different staging of MIBC, such as T2, T3, or T4N0M0 disease, respectively. As far as we know, our research is the first meta-analysis to assess the effect of NAC plus RC in patients with MIBC stratified by cancer staging.

Our study demonstrated that OS was not improved in patients with cT2N0M0 MIBC when NAC was combined with RC. The pooled results from the study by Sherif et al., a summary of two Nordic studies, published in the European Urology draw a conclusion of a HR of 0.85 for OS in cT2 disease but without statistical significance [11]. Although the JCOG0209 study found that NAC was superior to RC only for cT2 stage, no significant difference was observed in cT2 disease [17]. The studies conducted by Grossman et al. and Wallace et al. using a conversion calculation have drawn the same conclusion as described above [15, 16]. Furthermore, a recent multicenter study from Japan found NAC would not significantly improve the survival of patients with cT2 stage [24]. An observational study that included only T2 results suggested that NAC unlikely improved cancer specific survival and OS outcomes for T2 BC [21]. The RCTs are needed to confirm the effect of NAC on the survival of MIBC without extravesical invasion.

We revealed that the pathological results of cT2 BC were significantly improved by NAC, referring to increase of the rates of pCR. Surprisingly, this result is contrary to the survival rate. This difference between the pathological benefits and OS results may be explained by the following reasons. NAC is routinely applied in locally advanced disease or unresectable tumor to decrease tumor load, for the purpose of creating a condition of complete resection of invasive organ or reduction of tumor residue. cT2 BC is confined to the muscularis and belongs to early disease with less lymphatic metastasis. Potential micrometastases in cT2 BC is very rare, which results in fewer survival benefits of NAC than radical surgery. At present, the current results are from subgroup analyses of large-scale RCT studies or retrospective cohort studies, which brings a potential selection bias that may cause the conclusion that is not consistent with the actual situation. In addition, the deficiency of prospective research on cT2 disease brings difficulties to meta-analysis.

We found that NAC improved the survival outcomes of cT3-4 disease, which is consistent with the results of previous studies that included all staging of MIBC. Sherif et al. [11] found a remarkable survival effect on extravesical invasion disease when the cT3 results were analyzed separately. Kubota et al. [24], a retrospective study from a multicenter in Japan, confirmed this conclusion. Chemotherapeutic drugs could kill the metastatic tumor cells outside the bladder, inhibiting the spread of the tumor. The JCOG0209 research observed no obvious survival benefit between arms of patients with cT2 or cT3–4 disease. The authors of this research believed that the results were inaccurate because the number of patients in the study was too small to reflect the expected benefits of NAC. The lack of participants is due to their refusal to participate in this experiment.

Recently, the reliable accuracy in staging applications have been shown in multiparametric MRI (mpMRI) for BC [40, 41]. It was by the Vesical Imaging-Reporting and Data System (VI-RADS) score that an accurate preoperative BC staging could be provided [42, 43]. Especially, VI-RADS score 5 is extremely dependable in identifying extravesical invasion [44]. In the future, locally advanced patients for cT3-4 stage can be quickly identified and further recommended for neoadjuvant chemotherapy before surgery.

Our meta-analysis had some limitations. Firstly, simultaneous analysis of prospective and retrospective studies can lead to the risk of methodological heterogeneity. Therefore, we used subgroup analysis to test the reliability of the results, so as to improve the accuracy and quality of the research. Secondly, only 15 studies met the criteria, 8 of which were retrospective. Too little research, especially the lack of prospective studies, will lead to a low level of evidence. Thirdly, the chemotherapy regimens and cycles were not unified, which caused heterogeneity. However, our meta-analysis is the first and most comprehensive analysis to explicitly evaluate the effect of NAC on different stages of MIBC. The main advantages of our meta-analysis are as follows. Firstly, unlike the previous study combining cT2, cT3, and cT4 disease as a group of NAC, we analyzed cT2 and cT3-4 staging separately. Secondly, we used HRs and 95% Cls to analyze the survival results. Because the time factor is taken into account in the statistical analysis, the results can well reflect the prognosis of the disease. Thirdly, radical radiotherapy can no longer be used as the first-line treatment of BC, as we excluded outdated studies using RT, which is different from Yin et al. [13]

## 5. Conclusion

The outcomes of the present meta-analysis show the conflicting conclusion that the use of NAC followed by RC remarkably improved the result of oncological outcomes for patients with cT2 staging. Nevertheless, better pCR did not bring a survival benefit as shown by OS. Moreover, for patients with cT3-4 staging, remarkable improvements in OS and pCR were observed when NAC plus RC was performed in comparison with RC alone. The most reasonable cycle and regimens of NAC were not concluded (usually from two cycles to three cycles; GC, MVAC, or other platinum-based combination). When NAC is available, physicians should comprehensively consider its benefits and side effects.

## Figures and Tables

**Table 1:** Characteristics and interventions of studies included in the meta-analysis.

Author	Year	Country	Study design	Cancer stage	NAC regimen	NAC cycles	Interventions	Number of NAC+RC	Number of RC only	Number of the whole sample size	Median follow-up (months)
Kubota [24]	2021	Japan	Retrospective multicenter	cT2 and cT3-4	Cisplatin-based	3 or 4 cycles	NAC followed by RC versus RC alone	83	178	261	23

Soria [31]	2021	Europe, Canada and the USA	Retrospective multicenter	cT2	Cisplatin-based combination therapy.	3 cycles	NAC followed by RC versus RC alone	316	303	619	18

Mazzone [21]	2019	SEER database	Retrospective multicenter	cT2	NA	NA	NAC followed by RC versus RC alone	1519	2459	3978	NA

Lane [20]	2019	SEER database	Retrospective multicenter	cT2 and cT3-4	Cisplatin-based	NA	NAC followed by RC versus RC alone	381	1505	1886	25

Russell [22]	2019	Sweden	Retrospective multicenter	cT2 and cT3-4	NA	NA	NAC followed by RC versus RC alone	216	216	432	22

Nitta [23]	2019	Japan	Retrospective multicenter	cT2 and cT3-4	GC, MVAC, carboplatin, or GN	NA	NAC followed by RC versus RC alone	69	71	140	54

Hermans [19]	2018	Netherlands	Retrospective multicenter	cT2 and cT3-4	NA	NA	NAC followed by RC versus RC alone	191	4164	4355	118

Kitamura [17]	2014	Japan	Prospective multicenter	cT2 and cT3-4	MVAC	2 cycles	NAC followed by RC versus RC alone	64	66	130	55

Osman [18]	2014	Egypt	Prospective multicenter	cT2 and cT3-4	GC	3 cycles	NAC followed by RC versus RC alone	30	30	60	36

Khaled [32]	2014	Egypt	Prospective multicenter	cT3	GC	3 cycles	NAC followed by RC versus RC alone	59	55	114	37

Rosenblatt [33]	2012	Northern Europe	Retrospective multicenter	cT2 and cT3	Cisplatin+doxorubicin or cisplatin+MTX	2 or 3 cycles	NAC followed by RT+RC or RC versus RT+RC or RC	225	224	449	60

Sherif [11]	2004	Northern Europe	Prospective multicenter	cT2 and cT3	Cisplatin+doxorubicin or cisplatin+MTX	2 or 3 cycles	NAC followed by RT+RC or RC versus RT+RC or RC	306	314	620	56

Grossman [16]	2003	U.S.	Prospective multicenter	cT2 and cT3-4	MVAC	3 cycles	NAC followed by RC versus RC alone	153	154	307	101
Sherif [34]	2002	Northern Europe	Prospective multicenter	cT2 and cT3-4	Cisplatin+doxorubicin or MTX	3 cycles	NAC followed by RC versus RC alone	155	154	309	64

Malmström [35]	1996	Northern Europe	Prospective multicenter	cT2 and cT3-4	Cisplatin+doxorubicin	2 cycles	NAC followed by RT+RC versus RT+RC	151	160	311	60

GC: gemcitabine and cisplatin; GN: gemcitabine and nedaplatin; MTX: methotrexate; MVAC: methotrexate, vinblastine sulfate, doxorubicin hydrochloride (Adriamycin), and cisplatin; NA: not available; NAC: neoadjuvant chemotherapy; RC: radical cystectomy; RT: radiotherapy.

## Data Availability

The datasets used and/or analyzed during the current study are available from the corresponding author upon reasonable request.

## Supplementary Materials

Supplementary Materials Supplemental Figure 1: risk of bias and bias summary. Supplemental Table 1: the Newcastle-Ottawa Quality Assessment scale for cohort studies.
