# Supplementary material for: Neoadjuvant Chemotherapy for Different Stages of Muscle-Invasive Bladder Cancer: A Systematic Review and Meta-analysis
Source: Dis Markers. 2022 Mar 2;2022:8493519. doi: 10.1155/2022/8493519 (PMC8906988; doi:10.1155/2022/8493519)
Supplement: Supplementary Materials — Supplemental Figure 1: risk of bias and bias summary. Supplemental Table 1: the Newcastle-Ottawa Quality Assessment scale for cohort studies. [file 8493519.f1.docx]

Supplementary material 1. Search strings.

PubMed

(((((((((("Urinary Bladder Neoplasms"[Mesh]) OR "Bladder Neoplasms"[Title/Abstract]) OR " Bladder Neoplasm"[Title/Abstract]) OR "Bladder Cancer"[Title/Abstract]) OR "Bladder Cancers"[Title/Abstract]) OR "Bladder tumor"[Title/Abstract]) OR "Bladder tumors"[Title/Abstract]) OR "Malignant Tumor of Urinary Bladder"[Title/Abstract]) OR "Cancer of Bladder"[Title/Abstract]) AND (((("Neoadjuvant Therapy"[Mesh]) OR (Neoadjuvant Therapy[Title/Abstract])) OR (Neoadjuvant Chemotherapy[Title/Abstract])) OR (Neoadjuvant Chemotherapies[Title/Abstract])))

EMBASE

('bladder cancer'/exp OR 'bladder cancer':ab,ti OR 'bladder cancers':ab,ti OR 'bladder neoplasm':ab,ti OR 'bladder neoplasms':ab,ti OR 'bladder tumor':ab,ti OR 'bladder tumors':ab,ti OR 'cancer of bladder':ab,ti) AND ('neoadjuvant chemotherapy'/exp OR 'neoadjuvant chemotherapy':ab,ti OR 'neoadjuvant chemotherapies':ab,ti)


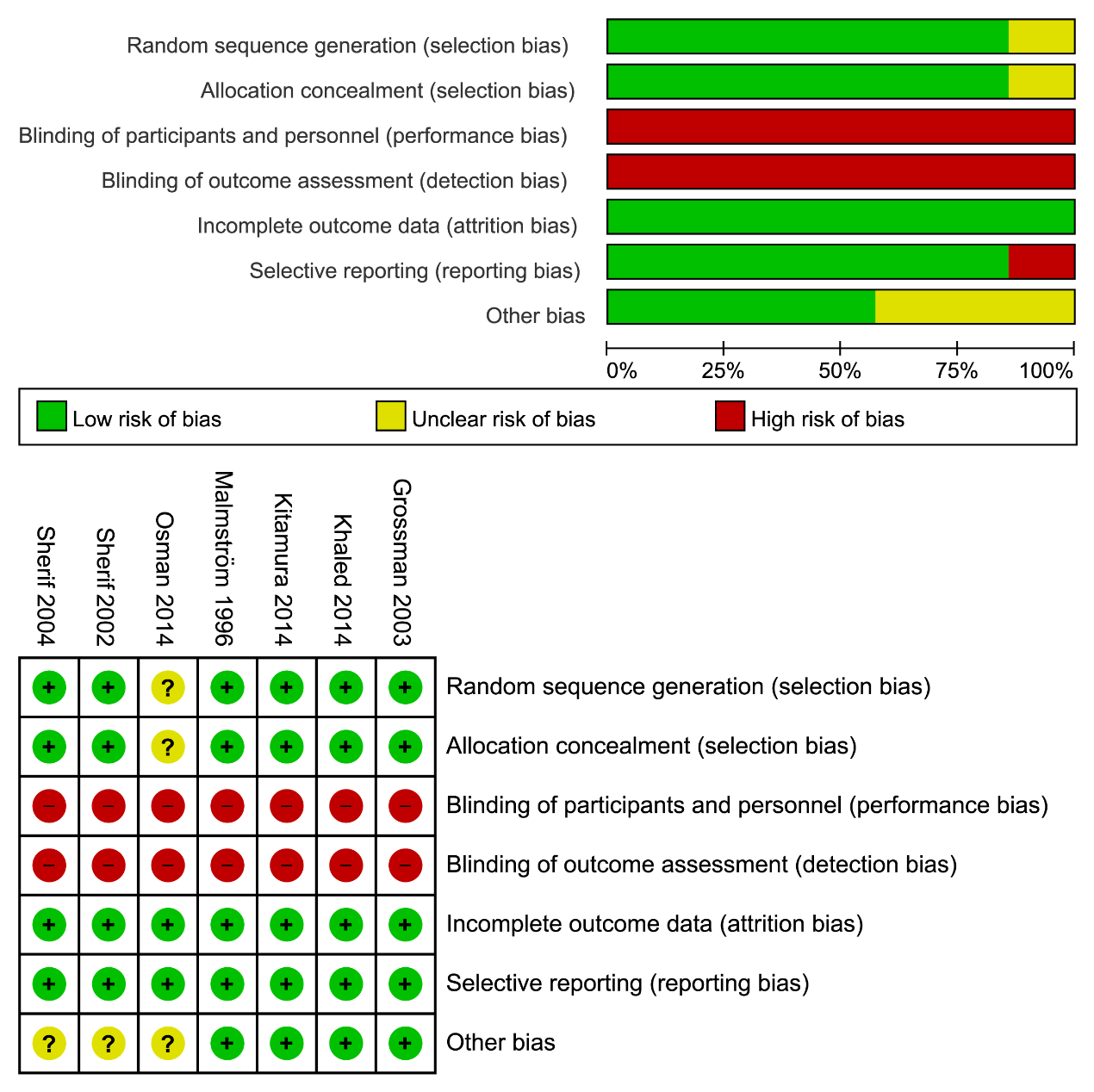


**Supplemental Figure 1: Risk of bias and bias summary**

**Supplemental Table 2: The Newcastle-Ottawa Quality Assessment scale for cohort studies**

| Study | Selection | Comparability | Outcomes | Total score |
| --- | --- | --- | --- | --- |
| Kubota et al.,2021 | ★★★★ | ★★ | ★★ | 8 |
| Soria et al.,2021 | ★★★★ | ★★ | ★★ | 8 |
| Mozzane et al.,2019 | ★★★★ | ★★ | ★ | 7 |
| Lane et al.,2019 | ★★★★ | ★★ | ★★ | 8 |
| Russell et al.,2019 | ★★★★ | ★★ | ★★ | 8 |
| Nitta et al.,2019 | ★★★★ | — | ★★★ | 7 |
| Hermans et al.,2018 | ★★★★ | — | ★★★ | 7 |
| Rosenblatt et al.,2012 | ★★★★ | — | ★★★ | 7 |
